# Colonic atresia and hirschsprung disease: a case report and review of the literature

**DOI:** 10.1186/s13256-023-03969-z

**Published:** 2023-06-07

**Authors:** Amirhossein Ladan, Reza Mahdian Jouybari, Mahnaz Zareh Akbari, Pegah Moharrami Yeganeh

**Affiliations:** 1 Department of Surgery, Ayatollah Mousavi Hospital, Zanjan University of Medical Sciences, Zanjan, Iran; 2 Department of Pediatrics, Ayatollah Mousavi Hospital, Zanjan University of Medical Sciences, Zanjan, Iran; 3 School of Medicine, Zanjan University of Medical Sciences, Zanjan, Iran

**Keywords:** Colon atresia, Hirschsprung disease, Case report, Newborn, Neontology

## Abstract

**Background:**

Colon atresia is one of the rarest congenital anomalies of the gastrointestinal tract, with an incident range of between 1 in 10,000 and 66,000 live births. Type I colonic atresia affects only the mucosal layer of the intestine and spares the intestinal wall and mesentery. Hirschsprung Disease is a rare association of Colon atresia and is usually diagnosed as a complication of atresia treatment.

**Case presentation:**

This study reports a 14-h term white middle-eastern female infant with type I transverse colonic atresia complicated by the association of Hirschsprung disease and provides a brief literature review of the topic. She presented with poor feeding, weakness, and failure to pass meconium, and her abdominal X-ray showed complete distal bowel obstruction. The presence of Hirschsprung disease was realized after complications of atresia surgery. The infant underwent a total of three surgeries involving an end-to-end anastomosis of the atresia, colostomy formation following anastomosis leakage, and Hirschsprung surgery. The patient ultimately expired.

**Conclusions:**

The association between colonic atresia and Hirschsprung disease poses a diagnostic and therapeutic challenge. Considering Hirschsprung disease as a possible association in colon atresia patients can facilitate proper decision-making in the course of treating colon atresia cases and achieving better outcomes.

## Background

Colon atresia is one of the rarest congenital anomalies of the gastrointestinal tract. Its frequency is estimated to be between 1 in 10,000 and 66,000 live births [1]. Most infants with colon atresia are term babies, and males have a slight predominance of prevalence [2, 3]. The etiology of this anomaly is thought to be of a vascular origin [1, 2, 4]. Clinical features include failure to pass meconium, abdominal distention, and bilious emesis [1]. Hirschsprung Disease is a rare association of Colon atresia and is usually diagnosed as a complication of atresia treatment [5]. The association between colonic atresia and Hirschsprung disease poses a diagnostic and therapeutic challenge. In this study, we report a female infant with colonic atresia complicated by the association of Hirschsprung disease and provide a brief literature review, aiming to contribute to the existing literature and knowledge around such cases and their proper diagnostic and therapeutic management.

## Case presentation

**Fig. 1:**
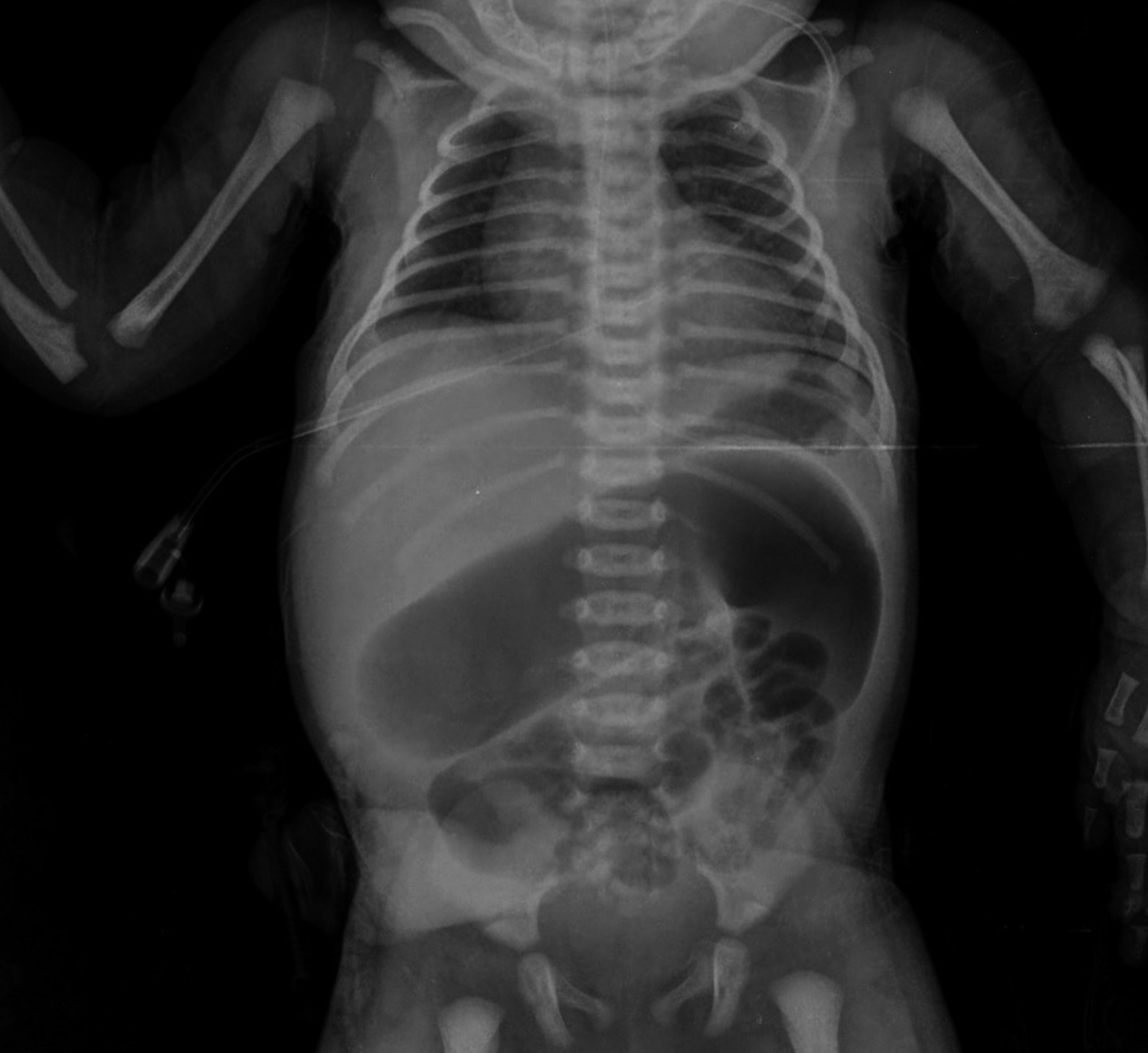
Plain radiograph of the patient shows gasless rectum and dilated intestines, evident of complete distal obstruction with multiple loops

A 3.6 kg term female middle-eastern neonate was delivered by cesarean section at 38 weeks of gestation with an Apgar score of nine at minute 1 and ten at minute 5 after birth. She was admitted to the NICU 14 h after birth due to poor feeding and weakness. In the initial examination, the abdomen was normal, no organomegaly was detected, and the rectum was open. The neonate failed to pass meconium during the first 48 h of life, and her abdomen gradually distended. She also developed bilious emesis and increased abdominal distention on the third day after birth. An abdominal ultrasound reported dilated stomach, intestinal loops, compressed large bowel loops, and free fluid. Figure 1 shows the abdominal radiograph of the patient.

**Fig. 2:**
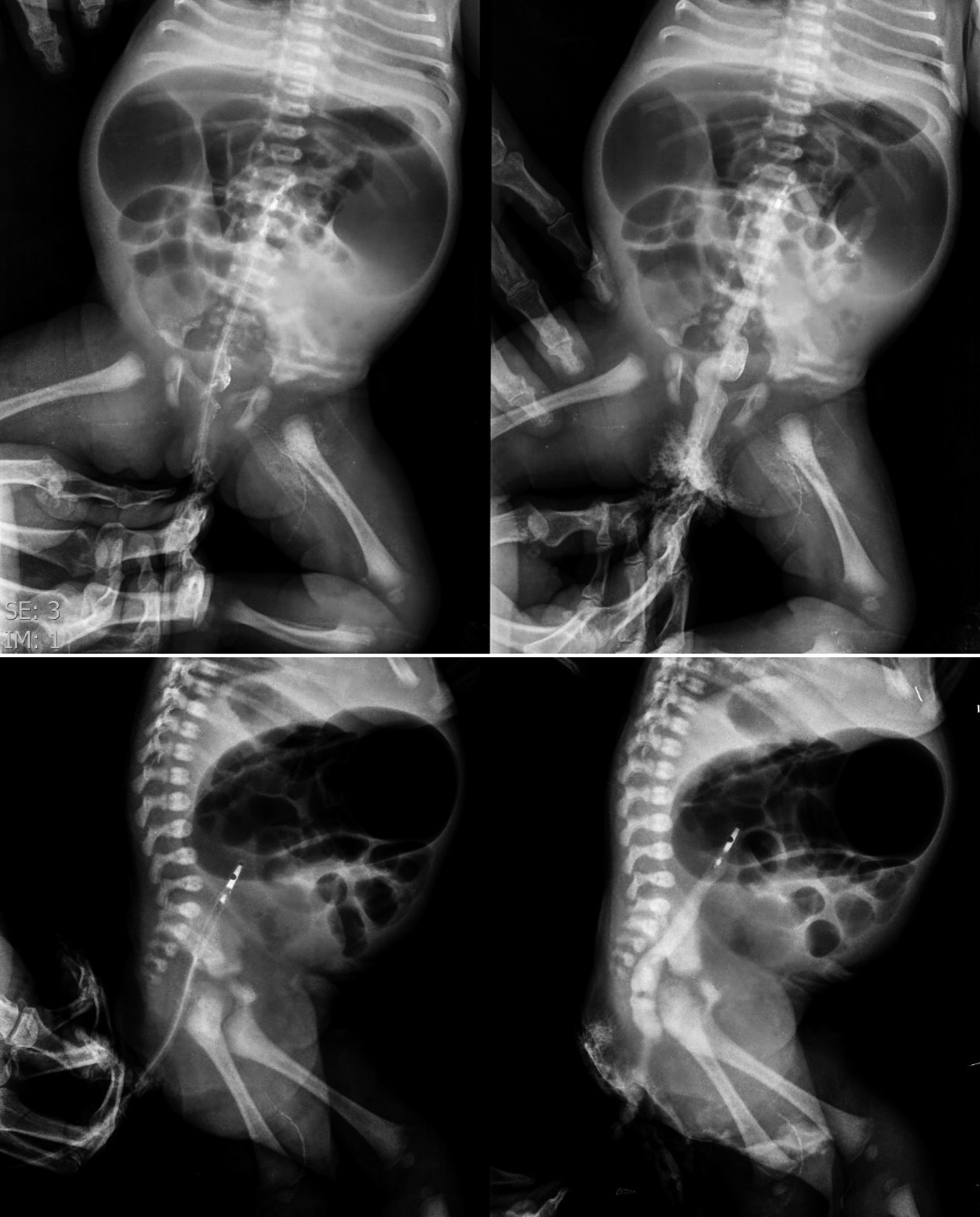
Gasrtografin enema showing contrast traveling limited to the splenic flexure

A gasrtografin enema was performed, revealing that the contrast's upward traveling was limited to the splenic flexure (Fig. 2). The neonate underwent laparotomy on the 4th day after birth, revealing type I transverse colon atresia in the splenic flexure. The transverse and ascending colon was deserosed and had formed a closed loop. The deserosed transverse and ascending colon was excised, and the ileum was connected to the descending colon through the end-to-end anastomosis. No evidence of other anomalies was detected in the patient. The patient passed stool after the surgery. On the third day after the surgery, the infant began to develop inflammation in the excision wound site and abdominal distention. She also developed bilious emesis and was unable to pass stool. She was taken back to the operating room and discovered that the anastomosis site was utterly disrupted. An ileostomy was formed, and biopsies of the distal colon were obtained, which came positive for Hirschsprung disease. The infant was scheduled for a Soave pull-through surgery at 41 days of age, which was completed uneventfully. The infant died at 45 days of age due to seizure and sepsis.

## Discussion and conclusion

This study reports a rare case of a newborn with type I transverse colon atresia that presented with poor feeding, weakness, and failure to pass meconium, which was later complicated by the occurrence of complications after a surgical attempt to restore the atresia and detection of associated Hirschsprung disease in the infant.

Colon atresia is categorized into three types [1, 6]:

- Type I, where the atresia only includes the mucosa and spares the intestinal wall and mesentery
- Type II, where a fibrous cord connects the atretic ends
- Type III, where there is a V-shaped mesenteric gap between the atretic ends. Subcategorized into Types IIIa and IIIb
  o IIIa: Containing only a mesenteric defect
  p IIIb: Known as "Apple peel deformity"
- Type IV, Where there are multiple atresias present.

The most common anatomical site affected by colonic atresia is the right colon, and the most common type of atresia is reported to be type III [7]. Type III atresia usually occurs proximal to the splenic flexure [8]. In contrast, types I and II seem to occur primarily distal to the splenic flexure [1], although type I has also been reported to occur throughout the colon length [8].

There are two widely performed surgical approaches to treating colonic atresia [7, 9]. The first one is performing a primary resection and anastomosis. The second treatment is forming a temporary stoma and repairing the intestinal continuity at a later stage. The staged approach is more favored in the literature as it allows for detecting possible associated anomalies [1, 10]. However, some authors prefer primary resection and anastomosis in favorable conditions [11, 12]. The difference in diameter between the two atretic ends and possible undiagnosed Hirschsprung disease associated with the atresia are the main challenges of primary anastomosis, potentially arousing post-operative complications and anastomosis failure [13]. Other surgical techniques such as end-to-side anastomosis with subsequent dehiscence, arthroplasty with side-to-side anastomosis, and diversion of both segments, have also been introduced [9]. A study in 2012 also proposed a "transanal approach" for two reported cases of rectal and low sigmoid atresia [14].

Hirschsprung disease, or congenital intestinal aganglionosis, is one of the most common causes of neonatal intestinal obstruction, with an incidence rate of about 1 in 5000 live births [15]. Few reported cases of the association of Hirschsprung disease with Colon atresia exist [16, 17]. The etiology of this association is unknown, but a couple of theories are trying to explain the phenomenon. One theory is that a pre-existing Hirschsprung disease may cause distension of the proximal ganglionic segment due to filling with meconium; This will then lead to intrauterine volvulus and induce atresia of the colon through the resulting vascular insufficiency [18]. Another idea is that the atresia prevents the migration of the ganglionic cells along the colon and results in Hirschsprung disease arising distally to the atresia [19]. A possibly common genetic cause of the two anomalies has also been proposed [13].

The mortality rate of colon atresia is around 10%[9, 20]. Delayed surgical intervention- especially after the first 72 h of life- increases the mortality rate dramatically (as high as 60%) [21, 22], mainly due to the formation of a closed blind loop between the ileocecal valve and the atretic end and the risk of rupture [1]. Delayed diagnosis of possible associated anomalies also increases mortality [5].

Although the association of Hirschsprung disease with Colon atresia is uncommon, obtaining rectal biopsies of detected colon atresia patients is advisable to check for Hirschsprung disease [10, 16]. Considering Hirschsprung disease as a possible association in colon atresia patients can facilitate proper decision-making in the course of treating colon atresia cases and achieving better outcomes. Moreover, assessing for meconium passing in newborn babies is essential to prevent delayed diagnosis of severe anomalies.

## Data Availability

Not applicable.
